# Investigating Patterns of Immune Interaction in Ovarian Cancer: Probing the O-glycoproteome by the Macrophage Galactose-Like C-Type Lectin (MGL)

**DOI:** 10.3390/cancers12102841

**Published:** 2020-10-01

**Authors:** Chiara Napoletano, Catharina Steentoff, Federico Battisti, Zilu Ye, Hassan Rahimi, Ilaria Grazia Zizzari, Marco Dionisi, Bruna Cerbelli, Federica Tomao, Deborah French, Giulia d’Amati, Pierluigi Benedetti Panici, Sergey Vakhrushev, Henrik Clausen, Marianna Nuti, Aurelia Rughetti

**Affiliations:** 1 Department of Experimental Medicine, “Sapienza” University of Rome, Viale Regina Elena 324, 00161 Rome, Italy; chiara.napoletano@uniroma1.it (C.N.); federico.battisti87@gmail.com (F.B.); hassan.rahimi@uniroma1.it (H.R.); ilaria.zizzari@uniroma1.it (I.G.Z.); marco.dionisi@uniroma1.it (M.D.); 2 Copenhagen Center for Glycomics, Department of Cellular and Molecular Medicine and School of Dentistry, Faculty of Health Sciences, University of Copenhagen, Blegdamsvej 3, DK-2200 Copenhagen, Denmark; steentoft@sund.ku.dk (C.S.); zilu@sund.ku.dk (Z.Y.); seva@sund.ku.dk (S.V.); hclau@sund.ku.dk (H.C.); 3 Department of Radiology, Oncology and Pathology, “Sapienza” University of Rome, Viale Regina Elena 324, 00161 Rome, Italy; bruna.cerbelli@uniroma1.it (B.C.); giulia.damati@uniroma1.it (G.d.); 4 Department of Gynecology-Obstetrics and Urology, “Sapienza” University of Rome, Viale Regina Elena, 324, 00161 Rome, Italy; federica.tomao@uniroma1.it (F.T.); pierluigi.benedettipanici@uniroma1.it (P.B.P.); 5 Department of Clinical and Molecular Medicine, “Sapienza” University of Rome, Via di Grottarossa 1035, 00189 Rome, Italy; deborah.french@uniroma1.it

**Keywords:** Epithelial ovarian cancer, MGL/CLEC10A/CD301, *O*-glycosylation, Tn antigen, cancer immunotherapy, DCs, glycan targeting

## Abstract

**Simple Summary:**

Changes in glycosylation occur during cancer transformation, altering the interaction among tumor, immune cells and microenvironment and impacting the anti-tumor immune response. The Macrophage galactose-like C-type lectin (MGL), expressed by dendritic cells (DCs), is the immune receptor recognizing the tumor Tn carbohydrate antigen. Recently, Tn/STn-CA125 glycoforms have been proposed as a promising tumor-associated biomarker and the targeting of MGL-Tn axis has been shown to be a valid therapeutic approach in ovarian cancer mouse model. Here we designed a chromatography method to identify glycoproteins relevant in DCs-tumor cell interaction by probing the ovarian cancer O-glycoproteome by means of MGL. The potential MGL binders identified were located on the cell membrane and in the intracellular compartment and matrisome, suggesting that MGL may play a role in sensing microenvironmental cues. These results may be relevant to investigate immune system-tumor interactions and contribute to the design of glycan targeting-based strategies for EOC immunotherapeutic interventions.

**Abstract:**

Glycosylation, the posttranslational linking of sugar molecules to proteins, is notoriously altered during tumor transformation. More specifically in carcinomas, GalNAc-type *O*-glycosylation, is characterized by biosynthetically immature truncated glycans present on the cancer cell surface, which profoundly impact anti-tumor immune recognition. The tumor-associated glycan pattern may thus be regarded as a biomarker of immune modulation. In epithelial ovarian cancer (EOC) there is a particular lack of specific biomarkers and molecular targets to aid early diagnosis and develop novel therapeutic interventions. The aim of this study was to investigate the ovarian cancer *O*-glycoproteome and identify tumor-associated glycoproteins relevant in tumor–dendritic cell (DC) interactions, mediated by macrophage galactose-like C type lectin (MGL), which recognizes the tumor-associated Tn *O*-glycan. Lectin weak affinity chromatography (LWAC) was employed to probe the *O*-glycopeptidome by MGL and *Vicia villosa* agglutinin (VVA) lectin using glycoengineered ovarian cancer cell lines and ovarian cancer tissues as input material. Biochemical and bioinformatics analysis gave information on the glycan arrangement recognized by MGL in tumor cells. The potential MGL binders identified were located, as expected, at the cell membrane, but also within the intracellular compartment and the matrisome, suggesting that MGL in vivo may play a complex role in sensing microenvironmental cues. The tumor glycoproteins binders for MGL may become relevant to characterize the interaction between the immune system and tumor progression and contribute to the design of glycan targeting-based strategies for EOC immunotherapeutic interventions.

## 1. Introduction

Epithelial ovarian cancer (EOC) still accounts for the highest mortality rate among all the gynecological malignancies [1]. Most of the patients (75%) are diagnosed at late stage of the disease (International Federation of Gynecology and Obstetrics (FIGO) stages IIIC and IV) and a high incidence of disease recurrence results in an overall survival of less than 20% at five years from diagnosis. Cytoreductive surgery and combined platinum–taxane chemotherapy comprise the standard therapy. In recent years, innovative treatments (target therapies and immunotherapeutic approaches) have been pursued with the aim to improve treatment efficacy and quality of life for the patients [2]. The increase in therapeutic options brings up the urgent need to identify novel biomarkers to orient the therapeutic choice and provide each patient with the best treatment [3]. Integrated bioinformatics analysis of omics databases has been performed to identify genes, expression profiles, and signaling pathways that can serve as potential biomarker candidates for progression and prognosis of EOC [4,5].

Changes in glycosylation arguably occur at early steps of carcinogenesis and have many physiological implications in the tumor microenvironment such as changes in oncogenic signaling and perturbation of cell-to-cell interactions, which in turn favor metastatic spread [6]. Furthermore, aberrant glycosylation profoundly affects tumor immunogenicity and interaction with immune cells, thus molding the anti-tumor immune response and immunosuppressive networks [7].

The aberrant expression of truncated *O*-glycans is the most common change in glycosylation present in epithelial cancer tissues. The truncation results in the expression of immature T (Galβ1-3GalNAcα1-*O*-Ser/Thr) and Tn (GalNAcα1-*O*-Ser/Thr) carbohydrate moieties and their sialylated versions, STn (NeuAcα2-6GalNAcα1-*O*-Ser/Thr) and ST (NeuAcα2-3Galβ1-3GalNAcα1-*O*-Ser/Thr) [6]. Multiple molecular mechanisms can account for the altered glycan biosynthesis: hypermethylation or somatic mutation of core 1, β3-Gal-T-specific molecular chaperone (COSMC), required for the elongation of mature *O*-glycans [8], or translocation of polypeptide GalNAc-transferases (GalNAc-Ts) from Golgi to endoplasmic reticulum (ER), that thereby inducing *O*-glycosylation of ER proteins [9]. 

Due to their tumor-restricted expression, Tn and STn glyco-epitopes have been exploited as diagnostic tools and immunotherapeutic targets for solid tumors [10]. 

In ovarian cancer, Tn/STn moieties are expressed and dysregulated expression of GalNAc-Ts has been found to correlate with increased tumor aggressiveness [11], while information on COSMC involvement is lacking. Furthermore, Tn/STn glycoprofiling of the circulating CA125 marker (MUC16 mucin) has been proposed as biomarkers for differential diagnosis of ovarian lesions [12,13].

C-type lectins are the immune receptors specialized for the recognition of glycan structures, associated with pathogens and transformed cells [14]. 

Macrophage galactose-like-C-type lectin (MGL) is the human lectin that recognizes the tumor-associated Tn glycan [15]. MGL, also known as CLEC10A/CD301, is expressed by macrophages and dendritic cells (DCs) [16] and plays an immunomodulatory role. Its targeting impacts DC metabolism [17], glycoantigen processing [18], and DC activation [19], and it has been proposed as a target for DC-based anticancer immunotherapy.

On the other hand, co-expression of MGL and Tn-ligand in tumors has been associated with a pro-tumoral immunosuppressive microenvironment [20,21].

In an ovarian cancer mouse model, MGL targeting by means of glycomimetic peptides was proven to activate DCs and effector immune cells, inducing tumor protection, also in combination with immune checkpoint inhibitor (ICI) therapy [22], suggesting that the MGL–Tn ligand axis may be relevant in ovarian cancer. Furthermore, in the era of ICIs, the identification of tumor ligands such as tumor glycoantigens, that are dynamically generated and interact with immune effector cells during tumor onset, may be relevant to increase ICI effectiveness [6,7].

The aim of the present study was to investigate the ovarian cancer *O*-glycoproteome in order to search for *O*-glycoproteins relevant in immune–tumor interactions mediated by MGL.

A lectin weak affinity chromatography (LWAC) method was developed, employing the recombinant hMGL (MGL–LWAC) and glycoengineered ovarian cancer cells and ovarian cancer tissues as input material. In parallel with MGL–LWAC, we used the well-established Tn-binding *Vicia villosa* agglutinin (VVA) lectin to capture the more broad VVA reactive ovarian cancer *O*-glycoproteome and compared our findings with the MGL dataset. 

The results provided information on the MGL binding requirements potentially occurring in vivo. MGL ligand candidates were identified by direct biochemical and bioinformatics analysis. Transmembrane and intracellular resident proteins and matrix components were consistently found to carry a Tn cluster motif and possibly to bind MGL. These results may contribute to our understanding of factors modulating the microenvironment in EOC and to the design of glyco-immunogens for DC-based cancer immunotherapy.

## 2. Materials and Methods

### 2.1. Recombinant MGL Protein

The recombinant human macrophage galactose-like C-type C lectin (rhMGL) receptor containing the extracellular portion of the human MGL (MGL_396-476_) linked to the human Fc of IgG_1_ was synthesized by GenScript USA Inc. (Piscataway, NJ, USA).

### 2.2. Cell Lines

OVCAR-3 SimpleCells (SC) were generated by zinc finger nuclease knockout (KO) of COSMC as previously described [23]. SKOV-3 SC were generated by CRISPR/Cas9 KO of COSMC with a validated gRNA [24] and screened by immunofluorescence as previously described [23]. The cells were cloned by limited dilution and the clones were analyzed by Indel Detection by Amplicon Analysis (IDAA) [25], followed by sequencing to define the induced mutation in the COSMC gene. SKOV-3 wild-type (WT) and SC were grown in McCoy’s 5a (modified) Medium (Life Technologies, Carlsbad, CA, USA) with 10% fetal calf serum (FCS) in the presence of 1% L-glutamine. OVCAR-3 WT and SC were grown into RPMI 1640 with 10% FCS in the presence of 1% L-glutamine and 4.5 g/L glucose. HEK 293 WT, SC, and STn positive cells, generated by knocking out (KO) of ST6GalNAc-I in HEK293 SC background [26], were cultured in Dulbecco’s Modified Eagle Medium (DMEM) medium with 10% FCS in the presence of 1% L-glutamine. 

### 2.3. Immunofluorescence

OVCAR-3 WT and SC, and SKOV-3 WT and SC, were grown on cover slips and fixed with 4% paraformaldehyde (PFA) for 10 min before immunofluorescence staining. Trypsinized HEK293 WT, SC, and STn cells were set on multiwall glass slides and fixed in ice-cold acetone for 7 min before immunofluorescence staining. Cells were then incubated with mouse monoclonal antibodies (MAb): anti-Tn (5F4 and 1E3 clones, undiluted hybridoma supernatant), anti-STn (3F1, TKH2, and B72.3 clones, undiluted hybridoma supernatant), and anti-T (3C9 clone, undiluted hybridoma supernatant) or biotin-coupled *Vicia villosa* agglutinin lectin (b-VVA, 0.4 µg/mL, Vector Labs, Burlingame, CA, USA) overnight (o/n) at 4 °C. For rhMGL staining, rhMGL (3 μg/mL) was added, followed by incubation with anti-hIgG-biotin (1:1000). 

Cells were washed and incubated with FITC-conjugated rabbit anti-mouse immunoglobulin (1:100) (Dako, Santa Clara, CA, USA) or Alexa 488-conjugate streptavidin (1:1000) (Invitrogen, Carlsbad, CA, USA) for 40 min in the dark at room temperature (RT), followed by mounting and DAPI staining (ProLong Gold Antifade, Thermo Fisher Scientific, Waltham, MA, USA). Cells were pre-treated with neuraminidase (0.1 U/mL in 0.1 M sodium acetate buffer, pH 5.5, *C. perfringens* neuraminidase type VI; Sigma Aldrich, St. Louis, MO, USA) for 1.5 h at 37 °C. Slides were visualized, and pictures were acquired using a Zeiss optical microscope.

### 2.4. Flow Cytometry

OVCAR-3 WT and SC, SKOV-3 WT and SC, and HEK 293 WT, SC, and STn ± neuraminidase treatment (0.1 U/mL in phosphate buffered saline (PBS) for 1.5 h at 37 °C) were analyzed by flow cytometry using rhMGL (3 μg/mL in PBS with 1% bovine serum albumin (BSA), 30 min at RT) or biotin conjugated VVA (0.5 μg/mL with 1% BSA, 30 min at RT). Cy3-conjugated anti-human IgG_1_ antibody (1:500; Sigma, St. Louis, MO, USA) and the Cy3-conjugate streptavidin (1:500) (Invitrogen, Carlsbad, CA, USA) were used as secondary antibodies. Cells were examined using a FACSAria II (Becton Dickinson, Franklin Lakes, NJ, USA) and data were analyzed using Flowing Software 2 (Turku Bioscience, Tykistökatu, Finland).

### 2.5. MGL–LWAC Column 

To prepare the MGL column for lectin weak affinity chromatography (LWAC), Protein G Sepharose 4 Fast flow beads (Life Technologies, Carlsbad, CA, USA) were packed into 0.9 m PFA tube (1/16” outer diameter, 0.03” inner diameter) (Upchurch Scientific, Oak Harbor, WA, USA) in Tris-buffered saline (TBS). rhMGL-Fc was injected into the column for binding with the beads and the MGL column was left to equilibrate at 4 °C over-night (o/n) in MGL binding buffer (TBS + 2 mM MgCl_2_ and 1 mM CaCl_2_). The ability of the MGL column to bind Tn/STn peptides was first tested by loading peptides corresponding to the amino acid sequence of IgA heavy chain (IgA-H) and podoplanin (PDPN) and their glycosylated versions (T/Tn) with defined glycosylation occupancy and eluting the column with 20 mM EDTA in TBS. The obtained fractions were then analyzed by MALDI-ToF MS (Autoflex, Bruker, Billerica, MA, USA), as previously described [27]. The peptide sequences are listed in Table S1. Glycopeptides were made by in vitro glycosylation as previously described [27].

### 2.6. Tumor Samples

Tumor samples from patients with high-grade serous ovarian carcinoma (OV1-OV4) were collected during surgery after informed consent was obtained (Ethics Committee of Policlinico Umberto I Hospital, C.E. Ref: 1454/24.07.08, Prot. no. 702/08), snap-frozen and stored at −80 °C until use for biochemical analysis. Tumor tissues for histopathology and histochemical analysis were embedded in paraffin (OV11–OV21). The information of the patients included in this study is reported in Table S2. 

### 2.7. Sample Preparation and Lectin Enrichment

Total cell lysate (TCL) and culture medium (secretome) from OVCAR-3 SC and SKOV-3 SC and TCL from HEK 293 SC and STn were processed as previously described [23]. Conditioned media was collected from cell cultures after 48–72 h. Dialyzed conditioned media was treated with neuraminidase to remove sialic acid (SA) (3 h at 37 °C). A short VVA agarose column (400 µL) was employed to enrich for glycopeptides. Elution was performed by heating the lectin (4 × 80 °C, 10 min) with 0.05% RapiGest.

TCL was obtained by the addition of 2 mL 0.05% RapiGest and sonication of cell pellets from one confluent T175 flask. After incubation with 5 mM DTT for 40 min at 60 °C, cell lysates and glycoprotein-enriched media were alkylated (10 mM iodoacetamide in the dark for 50 min), and digested with trypsin (25 μg o/n at 37 °C). Then, peptides and glycopeptides were purified by C18 Sep-Pak, treated with neuraminidase (except for HEK 293 STn TCL), and diluted in lectin-binding buffer. Lectin chromatography was performed as previously described for VVA [26] and as described above for MGL. When employing the rhMGL as catcher, a 0.9 m-long MGL column was set up (1.2 mg of rhMGL). Protease-digested TCL and secretome (160 mL starting material for each run) were loaded on the MGL column in MGL binding buffer. The MGL flow-throughs of three different 160 mL runs of the OVCAR-3 SC or SKOV-3 SC secretome were pooled together and passed on a 2.6 m long VVA agarose column. Only one MGL flow-through of the OVCAR-3 SC or SKOV-3 SC TCL was passed on the 2.6 m column. Enriched glycopeptides were desalted by StageTips and submitted for MS analysis.

For biochemical analysis, tumor tissues were mechanically pulverized with a mortar and pestle and then homogenized in the presence of 0.5% RapiGest. After sonication and washing, each sample was reduced and alkylated, digested with trypsin, and then purified by C18 Sep-Pak. Two tumor samples (OV1-OV2) were run in a 1.3 m long MGL column (2.4 mg of rhMGL) and the flow-throughs were run in a 2.6 m long VVA agarose column after neuraminidase treatment and C18 Sep-Pak purification. The other 2 tumor samples (OV3 and OV4) were treated with neuraminidase before the reduction step and were loaded directly into the VVA column. All the MGL and VVA columns elution fractions were desalted by StageTips and submitted for MS analysis.

### 2.8. Mass Spectrometry and Data Analysis

EASY-nLC 1000 UHPLC (Thermo Scientific, Waltham, MA, USA) interfaced via Nanospray Flex ion source to an Orbitrap Fusion mass spectrometer (Thermo Scientific, Waltham, MA, USA) was used for glycoproteomics analysis. In one duty cycle, the top 5 precursors were isolated and fragmented in HCD and ETD mode separately. A precursor MS scan (*m/z* 355–1700) of intact peptides was acquired in the Orbitrap at a nominal resolution setting of 120,000. The 5 most abundant multiply charged precursor ions in the MS spectrum at a minimum MS signal threshold of 100,000 were triggered for sequential Orbitrap HCD–MS/MS and ETD–MS/MS. MS/MS spectra were acquired at a resolution of 60,000. The activation times were 30 and 200 ms for HCD and ETD (ETciD) fragmentation, respectively, isolation width was 3 mass units for ETD and 1.6 mass units for HCD, and 1 microscan was collected for each spectrum. Supplemental activation (25%) of the charge-reduced species was used in the ETD analysis to improve fragmentation. Dynamic exclusion for 20 s was used to prevent repeated analysis of the same components. The mass spectrometry glycoproteomics data have been deposited in the ProteomeXchange Consortium [28] via the PRIDE partner repository with the dataset identifier PXD017510. All raw data were processed by Proteome Discoverer 1.4 software (Thermo Scientific, Waltham, MA, USA) using Sequest HT Node as previously described [29]. Briefly, all spectra were initially searched with trypsin full-cleavage specificity, filtered according to confidence level (medium, low, and unassigned), and further searched with the semi-specific enzymatic cleavage. The maximum number of missed cleavage sites was set to 2. In all cases, the precursor mass tolerance was set to 6 ppm and fragment ion mass tolerance to 20 mmu. Carbamidomethylation on cysteine residues was used as a fixed modification. Methionine oxidation and HexNAc attachment to serine, threonine, and tyrosine were used as variable modifications for ETD MS2. All HCD MS2 was pre-processed as described [23] and searched under the same conditions mentioned above using only methionine oxidation as variable modification. All spectra were searched against a concatenated forward/reverse human-specific database (UniProt, January 2013, containing 20,232 canonical entries and another 251 common contaminants) using a target false discovery rate (FDR) of 1%. FDR was calculated using target decoy PSM validator node. The resulting list was filtered to include only peptides with glycosylation as a modification.

### 2.9. Immunohistochemistry

Serial formalin-fixed, paraffin-embedded high-grade serous ovarian cancer tissue sections were deparaffinized in xylene, followed by absolute ethanol, 95% ethanol, and distilled water. Cancer patients’ characteristics are reported in Table S2. Before immunostaining procedures, sections were incubated in citrate buffer (pH 6.0) in a pressure cooker at 112 °C for antigen retrieval. Endogenous peroxidase activity was quenched by treatment with 3% H_2_O_2_. After blocking of nonspecific sites, the sections were incubated with the following antibodies: mAb anti-insulin-like growth factor binding protein 7 (IGFBP7) (H-3 clone, 1:100, o/n at 4 °C; Santa Cruz, Heidelberg, Germany), mAb 5F4 anti-Tn; mAb 3F1 anti-STn; rabbit polyclonal anti-nucleobindin-1 (dilution 1:200, 2 h at RT; AbCam) and rabbit polyclonal anti-endoplasmic reticulum resident protein 44 (ERp44) (15 μg/mL, o/n at 4 °C; Thermo Fisher Scientific, Waltham, MA, USA). Markers were visualized using the Dako EnVision+ System horseradish peroxidase (HRP)-labelled polymer kit (Dako, Santa Clara, CA, USA) for use with rabbit primary antibodies or UltraTek Horseradish Peroxidase (HPR) (ScyTek Laboratories, West Logan, UT, USA) for use with mouse primary antibodies, following the manufacturer’s instructions. The tissue sections were counterstained with hematoxylin (ScyTek Laboratories). The images were acquired with an Olympus BX51 microscope (Olympus Europe SE and Co. KG, Hamburg, Germany) and analyzed with IAS software (Rehlingen-Siersburg, Germany). Negative control slides were incubated with MOPC21 mAb (AbCam) as isotype control. Three independent investigators evaluated all specimens. 

## 3. Results

### 3.1. rhMGL Specifically Binds Tn Carrying Glycopeptides in Lectin Weak Affinity Chromatography (LWAC) Column

A lectin weak affinity column chromatography approach was set up to identify ovarian tumor glycoproteins binders for MGL and relevant in tumor–DCs interactions. 

**Figure 1:**
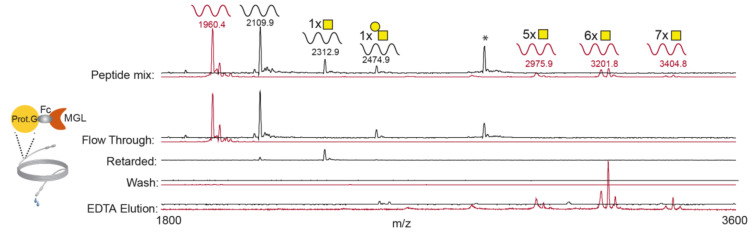
Macrophage galactose-like C-type lectin (MGL)-based lectin weak affinity chromatography (LWAC). MS spectra of IgA heavy chain (IgA-H; red) and podoplanin (PDPN; black) peptides and glycopeptide mixture employed: naked peptides (IgA-H and PDPN, respectively), Tn-glycopeptides carrying different amounts of Tn residues (IgA-H with 5, 6, or 7 Tn-residues) and T-(1T- PDPN) and Tn-(1Tn- PDPN) carrying peptides. MS spectra of distinct chromatography fractions are reported. * unknown impurity. Peptide sequences are listed in Table S1.

The human recombinant MGL-Fc chimera protein (rhMGL), containing the MGL carbohydrate recognition domain was immobilized onto Protein G Sepharose beads and packed in a narrow column (0.9 m long; Figure 1). Based on previous studies predicting the preferential binding of MGL to Tn carbohydrate residues [30], the performance of the MGL column was evaluated employing two unglycosylated peptides (IgA-H and PDPN) and their corresponding T and Tn-carrying glycopeptides. The peptides (Table S1) were selected in order to represent different peptide sequences and different glycan occupancy and structure. The peptide mixture was loaded through the MGL–LWAC and the fractions were collected and analyzed by MALDI-ToF MS (Figure 1).

Unglycosylated and T glycopeptides were found in the flow-through (FT) fraction, while the glycopeptide carrying 1Tn was found in the retarded fraction (obtained by washing the column with TBS after loading). No peptide was detected following subsequent washing of the column (Wash).

The addition of EDTA, which abrogates the MGL–ligand interaction by sequestering Ca^++^, allowed the exclusive elution of the Tn glycopeptides (carrying 5 to 7 Tn carbohydrates). These results were also confirmed employing other Tn-glycopeptides with different sequences (data not shown), suggesting that peptides carrying multiple Tn were selectively isolated by the MGL–LWAC chromatography.

As some studies have proposed that MGL may also recognize the STn structure [31], a mixture of STn-glycopeptides, partially or fully sialylated, was loaded onto the MGL–LWAC column. The result indicates that fully sialylated STn-glycopeptides, independent of number of glycosylation sites, did not bind the MGL–LWAC column and were released in the FT. Partially sialylated glycopeptides, in which Tn residues were still available, were found in the EDTA eluted fraction and in the FT (Figure S1a,b).

These results suggest that MGL preferentially binds multiple GalNAc residues and that the presence of sialic acid (SA) reduces or even abrogates MGL binding.

### 3.2. rhMGL Binds Tumor Glycan Tn on Ovarian Cancer Cells

**Figure 2:**
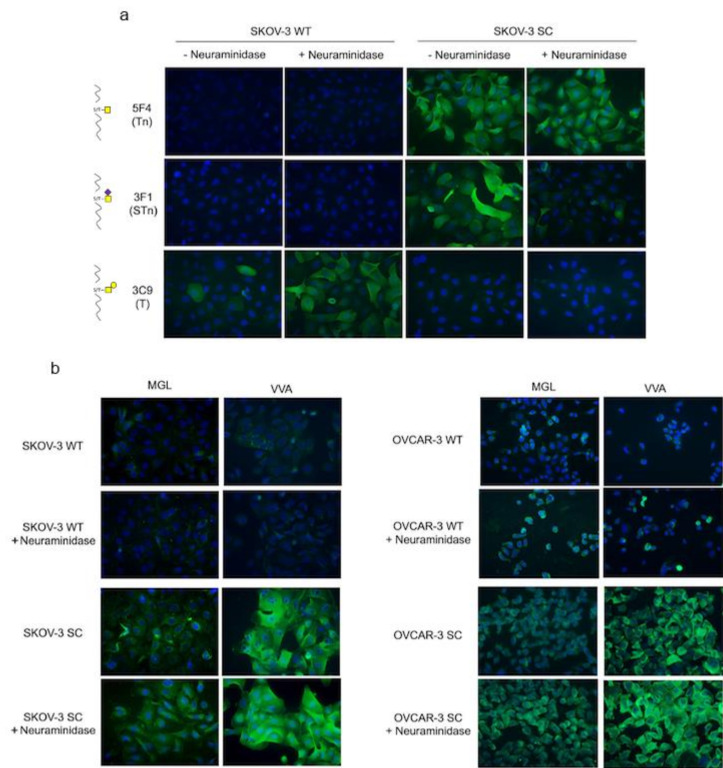
Glycoprofiling and lectin binding of SKOV-3 and OVCAR-3 ovarian cancer cell lines. (**a**) Immunofluorescence of wild-type (WT) and SimpleCell (SC) modified SKOV-3 cell line with and without neuraminidase treatment stained with anti-Tn mAb 5F4, anti-STn, mAb 3F1, and anti-T mAb 3C9. (**b**) Fluorescence microscopy of WT and SC SKOV-3 and OVCAR-3 with and without neuraminidase treatment, stained with rhMGL or VVA lectin (magnification 20×).

Ovarian cancer cell lines were glycoengineered to maximize expression of truncated Tn and STn glycoantigens and used as a source of MGL ligands for MGL–LWAC. We previously established and characterized the OVCAR-3 SC line expressing high levels of Tn and STn [23]. Here, we additionally used CRISPR/Cas9 to knock out COSMC in ovarian SKOV-3 cancer cells (Figure S2a). Glycoprofiling using carbohydrate-specific mAbs, with and without neuraminidase treatment, showed that the parental SKOV-3 cells displayed high levels of ST glycans and some T, while Tn and STn were undetectable. On the contrary, the isogenic SKOV-3 simple cell (SKOV-3 SC) displayed high levels of Tn and STn and the absence of ST and T structures (Figure 2a).

The MGL binding specificity for Tn/STn glycans was tested in OVCAR-3 SC and SKOV-3 SC cell lines by immunofluorescence. rhMGL binding occurred in both cell lines and was enhanced by neuraminidase treatment, while little or no binding occurred in the parental cell lines, even following neuraminidase treatment (Figure 2b). In all cell samples, MGL binding appeared weaker than the binding of VVA lectin, which selectively and strongly recognizes Tn residues. These results were also assessed by flow cytometry (Figure S2b) and it was collectively confirmed that rhMGL binds to cells expressing Tn-glycoforms and the binding is enhanced by the removal of SA.

This evidence was confirmed by testing MGL binding to HEK293 cell line variants [26]: the HEK293 SC, in which COSMC was deleted by genome editing, and HEK293 STn, a ST6GalNAc-1 KI clone (Figure S1c). As before, rhMGL weakly bound to HEK293 SC, while VVA displayed stronger binding. Both MGL and VVA binding was completely abolished in HEK293 STn cells displaying high levels of sialylation (Figure S1c), while it was restored following neuraminidase treatment (Figure S1d), thus supporting the hypothesis that sialylation hinders MGL binding.

### 3.3. MGL Preferentially Recognizes Ovarian Cancer Associated Glycoproteins Carrying Multiple Tn Carbohydrate Moieties Clustered on Adjacent Amino Acid Sites

The LWAC approach has successfully been employed to identify serum glycoproteins in gastric cancer patients using VVA lectin as catcher [32]. The *O*-glycoproteome of both OVCAR-3 SC and SKOV-3 SC cells was investigated by MGL-or VVA–LWAC, and the total cell lysate (TCL) and conditioned cell culture medium (secretome) were analyzed.

**Figure 3:**
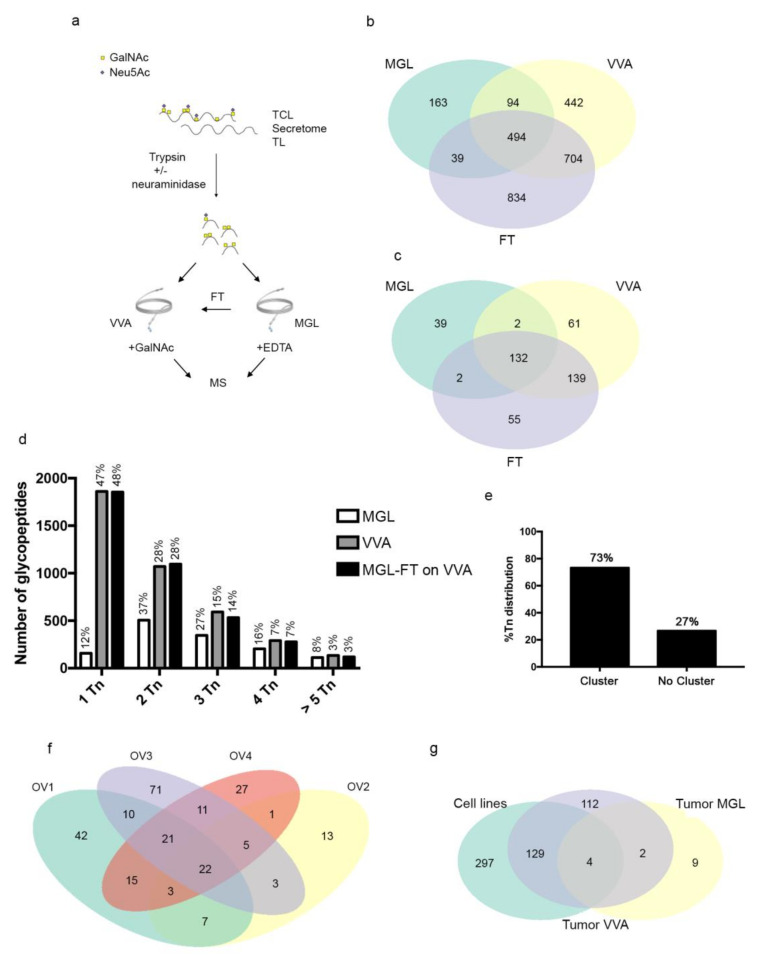
*O*-glycoproteome analysis of ovarian cancer cells and tissues by LWAC. (**a**) Schematic depiction of LWAC strategy to identify the *O*-glycoproteome of SKOV-3 Simple Cells (SC) and OVCAR-3 SC ovarian cancer cell lines (total cell lysates (TCL) and secretome and tumor cell lysates (TL)). (**b**,**c**) Venn diagrams showing distribution of Tn-glycopeptides (**b**) and Tn-glycoproteins (**c**) identified by rhMGL- or VVA–LWAC. FT refers to samples first enriched by MGL–LWAC followed by VVA–LWAC enrichment on the flow-through. (**d**) Histograms representing the accumulated total number of glycopeptides carrying different moles of Tn-glycans isolated from TCL and secretome of ovarian cancer cell lines by rhMGL (white bars), VVA (gray bars), and the flow-through of MGL–LWAC analyzed by VVA–LWAC (black bars). Values on top of histograms represent the percentage of the total Tn-carrying glycopeptides identified in the three experimental settings. (**e**) Histograms reporting the percentage of MGL peptide binders carrying Tn-residues clustered on adjacent amino acids (cluster) or present in isolated sites (no cluster). (**f**) Venn diagrams showing distribution of Tn-glycoproteins isolated from four ovarian cancer tumor samples by rhMGL- or VVA–LWAC. (**g**) Venn diagrams showing distribution of Tn-glycoproteins from tumor cells and ovarian cancer cell lines identified by rhMGL- or VVA–LWAC.

Samples were neuraminidase treated to enhance exposure of Tn glycans and trypsinized, as we previously observed improved enrichment by applying glycopeptides instead of glycoproteins [32]. The samples were loaded onto a MGL- or VVA–LWAC column, then eluted by EDTA or GalNAc, respectively. In addition, the FT fraction from the MGL–LWAC column was loaded onto the VVA–LWAC column to isolate those Tn-carriers not bound by MGL (Figure 3a). While our study was by no means quantitative, the analysis indicated that the cell lysates were richer sources of Tn-glycopeptides and glycoproteins than secretome in both SKOV-3 SC and OVCAR-3 SC cells, at least with the presented method (Table S3), as we have also previously observed [33]. 

In the dataset from ovarian SC cancer cells, a fraction of the *O*-glycopeptide repertoire (29%, 790/2770) was recognized by MGL and a subset of those binders was shared with VVA (627/790). As expected, VVA recognized the vast majority of the *O*-glycopeptide identified repertoire (94%, 2607/2770) (Figure 3b). 

From this analysis, 430 unique glycoproteins were identified: 91% were VVA binders (391/430) and 41% were recognized by MGL, and again, a subset was shared between MGL and VVA (136/175) (Figure 3c), suggesting that MGL displays more constricted carbohydrate recognition in ovarian cancer cell models compared to VVA. 

This result prompted us to verify whether differences between the distinct subsets of binders (MGL vs. VVA) could be identified. MGL preferentially bound glycopeptides carrying a minimum of two Tn, while VVA had a preference for 1Tn-glycopeptides (Figure 3d), as previously reported [23]. Interestingly, the glycan density as well as glycan arrangement along the peptide backbone appeared to be peculiar for MGL binding: 73% of MGL binders we identified carried Tn moieties clustered on adjacent amino acid residues, suggesting that this Tn distribution is a distinctive molecular trait of MGL binders likely reflected in vivo as well (Figure 3e). 

### 3.4. MGL Recognizes Intracellular and Extracellular Glycoproteins in Ovarian Tumor Tissues

We extended the search of putative MGL ligands in tumors, probing the *O*-glycoproteome of EOC samples by VVA- and MGL–LWAC. Tumor biopsies of high-grade serous ovarian carcinoma (FIGO stage IIIC) from untreated patients were processed and analyzed (Table S2). 

The OV4 and OV3 tissue samples were homogenized, neuraminidase treated, and trypsinized, then loaded by the VVA–LWAC chromatography column: 543 and 250 glycopeptides were identified, respectively, corresponding to 148 and 105 unique glycoproteins (Table S4; Figure S3a).

When ovarian tissue samples (OV1, 2) were analyzed by MGL–LWAC, neuraminidase treatment was avoided to preserve the tumor glycan repertoire. The analysis resulted in a low yield: glycopeptides corresponding to 15 unique glycoproteins were isolated from the tissue samples (Table S4). An extracted ion chromatogram showing the glycopeptide enrichment with MGL for OV2 is included in Figure S4a. When the FT fractions were subjected to neuraminidase treatment and analyzed by VVA–LWAC chromatography, 138 unique Tn glycoproteins were identified (Figure S3b), suggesting that OV1 and OV2 tumors displayed high levels of sialylation that could reduce Tn moieties’ availability to MGL binding. Indeed, more than 85% of all identified glycopeptides were suggested to be modified with GalNAc moieties, attributed to MS2 with the oxonium ions used to distinguish between GlcNAc and GalNAc (Figure S4b) [34].

**Figure 4:**
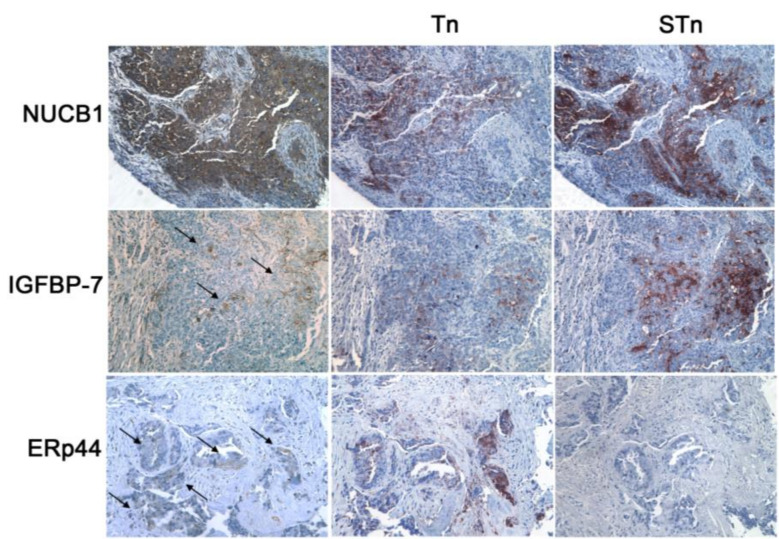
Tissue staining patterns of tumor MGL binders. Expression of nucleobindin-1 (NUCB1), insulin-like growth factor binding protein 7 (IGFBP-7), and endoplasmic reticulum resident protein 44 (ERp44) was evaluated by immunohistochemistry in serial tissue sections of high-grade serous ovarian carcinoma. In parallel, Tn and STn expression was assayed (60× magnification).

The analysis of the *O*-glycoproteome of ovarian tumor samples indicated that out the 256 unique *O*-glycosylated proteins isolated by LWAC, irrespective of lectin binding, 22 (9%) were consistently found in every tumor sample (Figure 3f). We further selected those tumor MGL binders that were also recognized by VVA in both tumor and cell lines (Figure 3g; Table S5), and, following manual validation of the glycopeptide mass spectra (Figure S5), three molecules were identified as MGL binders: insulin-like growth factor binding protein 7 (IGFBP7), endoplasmic reticulum resident protein 44 (ERp44), and nucleobindin-1 (NUCB1). These glycoantigens were expressed with distinct intensity and patterns in ovarian tumor samples, as detected by immunohistochemistry on serial tissue sections (Table 1) in tumor areas positive for Tn and STn antigens (Figure 4). NUCB1 was expressed in almost all the samples tested (10/11, with low/intermediate intensity in the majority of tumor cells). When present, IGFBP7 expression had a strong intensity and was restricted to clusters of cancer cells (6/11 samples). The chaperon ERp44 protein was also detected with a weak intensity, as expected for an ER resident protein, in 36% of the tumors (4/11). Tn and STn displayed high and heterogeneous expression among the tumors, with a prevalence of the STn carbohydrate antigen.

### 3.5. The MGL Binding Motif Is Carried by Surface, Intracellular and Extracellular Matrix Glycoproteins

The complex glycosylation profile and sialylation levels may be factors limiting enzymatic digestion, thus leading to underestimation of lectin binders [35]. We had previously observed an underrepresentation of glycopeptides from tandem repeats of mucins including MUC1 using the VVA–LWAC strategy [23]. Our data thus showed that MGL recognized tumor glycoproteins with intracellular distribution in ovarian tumors, while other MGL ligands such as MUC1 were not detected by MGL–LWAC. 

This prompted us to analyze the ovarian cancer glycoproteome employing as criteria the presence of Tn moieties clustered on adjacent amino acids that appeared to be preferentially recognized by MGL (Figure 3e). A two-step analysis was performed: first we selected the glycoproteins carrying the Tn cluster found in the ovarian tumor samples, irrespective of the lectin binding, and 52 tumor glycoproteins were identified (Table S4). Within this subset, we then refined the search to those glycoproteins that were also found in the ovarian SC dataset with the same Tn cluster motif. Table 2 reports these glycoproteins and the corresponding glycosites consistently found in tumors and SC cell lines.

From this analysis, the mucins MUC1, MUC16, and MUC24 were found to carry the Tn cluster. Few glycosites were detected for those mucins again, confirming that complex glycosylated molecules are unsuitable substrates for enzymatic digestion. In addition, podocalyxin (PODXN) and nidogen 2 (NID-2), which are cell surface glycoproteins involved in cell adhesion and invasion, were decorated by a Tn cluster.

Several components of the extracellular matrix were identified in this analysis: proteoglycans including dystroglycan 1 (DAG1), syndecan-3 (SDC3), fibronectin 1 (FN1), versican core protein (VCAN) and agrin (AGRN).

ERp44, which we have shown to be directly recognized by MGL, was again validated as a putative MGL ligand, and other intracellular proteins, mostly resident in the ER compartment, were shown to carry the Tn cluster: lysosome-associated membrane glycoprotein 1 and 2 (LAMP1, LAMP2), sulphydryl oxidase 1 (QSOX1), and protein sel-1 homolog 1 (SEL1). In addition, leucin-rich repeated-containing protein 8D (LRR8CD), involved in the regulation of cell metabolism, was decorated with a Tn cluster. 

Interestingly, most of the Tn clusters were flanked by other Tn moieties. These results suggest that ovarian tumor glycoproteins localized on the cell surface and intracellular compartment and in the matrix carry the Tn cluster recognized by MGL. 

## 4. Discussion

The type and location of the immune infiltrate present at the tumor bed are critical factors associated with the outcome of the disease and crucial for the choice of therapeutic options. In EOC, tumor infiltration of CD8^+^ T cells and mature DCs are immune biomarkers associated with a favorable prognosis [36,37]. Immunotherapeutic approaches such as peptide/DC based vaccinations or infusion of ex-vivo expanded tumor infiltrating lymphocytes (TILs) can activate anti-tumor immunity, with partial therapeutic benefit for EOC patients [38,39,40]. On the other hand, targeting the immune checkpoint blockade has not given the expected results, with low rates of response [41]. It is thus crucial to dissect the mechanisms for recruiting and trafficking immune cells from and to the ovarian tumor microenvironment in order to unleash and boost existing anti-tumor immunity in order to obtain therapeutic efficacy. In this work, we attempted to identify ovarian glycoproteins potentially relevant in tumor–DCs interactions by probing the ovarian cancer *O*-glycoproteome with MGL C-type lectin and VVA lectin. We chose MGL lectin for its role in tumor immunity and its selective recognition of the tumor-associated Tn glycan. We also included the better established VVA–LWAC approach, successfully employed to identify tumor serum Tn-biomarkers [32], to get a more complete picture of the ovarian cancer *O*-glycoproteome.

MGL–LWAC exclusively retained glycopeptides carrying Tn moieties, while naked peptides or peptides decorated with different glycan patterns (T, STn) were not bound, indicating that hMGL maintained its binding specificity. This method is distinguishable from a previous strategy using hMGL-conjugated beads in a pull-down assay for identifying glycoproteins in cancer cell models [42,43]. MGL–LWAC allowed us to enrich for glycopeptides, running in a long and narrow column, and not glycoproteins, avoiding the possibility of isolating other molecules co-purifying with the actual MGL ligand and mapping the sites for *O*-glycosylation. 

As input material, we employed SC cancer cell lines and EOC tissue samples. In the SC ovarian cancer cells, the COSMC gene was deleted to enhance the expression of Tn/STn truncated *O*-glycans, as confirmed by immune-glyco profiling. This experimental model maximizes the aberrant Tn/STn expression observed in tumors and is a valid tool to identify *O*-glycosylation substrates and unequivocally map *O*-glycosites that are likely to occur in vivo. 

MGL and VVA displayed distinct reactivity profiles, although they recognized the same glycan moiety. MGL displayed a more selective staining of SC cell lines than VVA, and proteomics analysis of SC ovarian cancer cells confirmed this observation. Glycopeptides identified by MGL–LWAC were less abundant than those obtained by VVA–LWAC analysis, and most of the MGL binders were also recognized by VVA. It is reasonable to speculate that the binding motif recognized by MGL is quite restricted because of the physiological role of this lectin and therefore few glycoproteins are able to efficaciously interact. Indeed MGL–Tn interactions have been related to the modulation of both activated and regulatory T cells [44,45]. 

In vitro, MGL preferentially binds densely glycosylated structures, as shown by several studies analyzing binding to glycopeptide libraries [30,46]. Furthermore, the STn carbohydrate has been proposed to act as a low-affinity ligand [31], however our results of binding assay and biochemical analysis strongly suggest that sialylation blocks or at least diminishes MGL-binding.

From the analysis of SC cell lines, we found that MGL preferentially recognized glycopeptides carrying Tn moieties clustered on adjacent amino acid residues. However, not all of the binders shared this molecular feature, suggesting that other factors such as glycopeptide conformation might influence the binding as well. In previous studies, we observed a high affinity of IgG auto-antibodies toward Tn-glycopeptides in cancer patients [47], and now we are starting to understand that these responses are highly glycopeptide selective (unpublished data). The connection of the restricted binding pattern of MGL with this phenomenon is of great interest for future investigation, in particular for future development of glycoimmunogens and vaccination studies.

When the *O*-glycoproteome of ovarian tumors was probed by means of MGL and VVA, a core of shared glycoproteins was identified, although only very few MGL-specific binders were found in the in vivo dataset. This is probably due to MGL’s more restricted binding pattern compared to VVA and the heterogeneous Tn/STn expression. We identified ERp44, NUCB1 and IGFBP7 as tumor Tn-glycoproteins ligands for MGL, also found in the *O*-glycoproteome of SC cell lines. These molecules were expressed in EOC tissue samples and co-localized with Tn antigen, as detected by immunohistochemistry. The expression variability of MGL binders did not appear to be ascribed to tumor histotype, since all samples tested were high-grade serous lesions, but probably to their biological role. IGFBP7 downregulation in HGSC tumors was associated with reduced overall survival and poor outcome, suggesting its involvement in the tumor suppression pathway [48]. Similarly, we found no or poor IGFBP7 expression in 8 of 11 samples tested.

So far, no role in EOC development has been shown for NUCB1 and ERp44, however it is interesting to note that the Ca^++^ binding protein NUCB1 has been shown to play a role in epithelial mesenchymal transition (ETM) signaling [49], which significantly contributes to cisplatin resistance in ovarian cancer cells [50]. 

Most recently ERp44, an ER resident protein involved in protein folding, has been shown to be a target for auto-antibodies in colorectal cancer patients and was proposed as a biomarker [51]. It is interesting to note that ERp44 was consistently identified in all tissues and samples tested by both MGL- and VVA–LWAC, despite its weak staining on tumor tissue sections, and further studies are warranted to investigate the role of ERp44 in EOC.

Immune interactions occur and trigger metabolic cell responses at low stoichiometric ligand/receptor ratios. It is reasonable to suppose that in vivo MGL engagement in DCs may also efficaciously occur in the presence of low levels of ligand, thus affecting the potency of DC antigen presentation. This is a crucial factor when planning immunotherapeutic strategies focused on activating adaptive immunity, whereas passive immunotherapy needs an expression threshold of the antigen target to be effective.

Due to the weak affinity of MGL, as discussed above, and to gain further information, we analyzed the *O*-glycoproteome of tumor samples, and selected those glycoproteins carrying the same Tn clusters, preferentially recognized by MGL, in both tumor and SC ovarian cancer cells.

Following this criterion, we identified glycoproteins already shown to be ligands for MGL in other experimental settings (PODXN, LCC8RD [43] and LAMP1 [42]) as well as the ERp44 molecule, indicating the validity of our approach to selecting putative MGL ligands and mapping the specific glycosites.

The tumor markers MUC1 and MUC16 were identified as glycoproteins carrying the Tn cluster. We have shown that Tn-MUC1 interacts with DCs through MGL engagement [18,52]. Tn/STn-MUC16 and MUC1 glycoforms are differently expressed in borderline and malignant serous ovarian tumors from benign lesions, suggesting this glycoform signature as a promising tumor-associated biomarker [53]. Furthermore, glycoprofiling of circulating CA125 appeared to improve the differential diagnosis in ovarian cancer [12,13].

Interestingly, molecules with intracellular and extracellular distribution were decorated with Tn clusters.

*O*-glycosylation of intracellular proteins is a trait of aberrant glycosylation observed in tumors, probably due to the retrograde relocation of GalNAc-Ts from the early Golgi cisternae to the ER compartment [54]. We found that LAMP2, QSOX1 and SEL1, in addition to ERp44 and LAMP1, were decorated with the Tn clusters, and all of them have ER/MHC II compartment distribution. Recently, it has been proposed that MGL could act as sensor of distress in a breast cancer model [55]. In combination with our observations, it may be hypothesized that MGL can sense these tumor intracellular Tn moieties as cell-damage molecular signals.

Components of the extracellular matrix were also found as MGL ligands in the ovarian tumor *O*-glycoproteome. FN1, SDC3, VCAN, NID-2, and AGRN were found to be decorated with Tn cluster motifs. Among these molecules, several have been shown to be relevant to ovarian cancer and proposed as biomarkers, including NID-2 and VCAN [56,57]. Most recently, VCAN has been included in a prognostic gene signature in high grade serous ovarian cancer [58]. Changes in the matrisome alter tumor adhesion properties, thus contributing to the spilling of cancer cells in the peritoneum and impacting the immune infiltrate at the tumor bed [59].

In early work, MGL was shown to modulate DCs trafficking into the lymphoid tissues interacting with Tn moieties expressed by the lymphatic endothelial cells [60]. For now, we can only speculate that MGL expressed by DCs may interact with the Tn ligand exposed by matrix components and contribute to tumor immunoinfiltration. 

From this point of view, the development of glycan-targeting strategies modulating the Tn-MGL-mediated interactions may be an appealing and innovative strategy to influence the immune cell tissue localization and the immune response [61].

## 5. Conclusions

In this study, we designed a novel LWAC method employing a recombinant MGL lectin to identify ovarian tumor-associated Tn-glycoproteins potentially able to interact with MGL in vivo. Our observations confirm that a Tn cluster motif is preferentially recognized by MGL. Among the potential MGL ligands, we identified glycoproteins that are linked to ovarian cancer progression, such as IGFBP7, MUC16, and VCAN, suggesting that MGL expressed by DCs may exert its immunoregulatory role by interacting with distinct cellular and matrix components. These observations require validation in a larger cohort of patients, and also in regard to the distinct histotype, molecular features and immune infiltration profile of ovarian cancers. We believe these results will contribute towards understanding tumor-immune cell interactions in EOC progression and designing novel immunotherapeutic interventions for EOC.

## Figures and Tables

**Table 1:** Expression of ovarian tumor glycoproteins binders of MGL.

Ovarian Cancer Patient	NUCB1		IGFBP7		ERP44		Tn		STn	
	% Positive Cancer Cells	Intensity	% Positive Cancer Cells	Intensity	% Positive Cancer Cells	Intensity	% Positive Cancer Cells	Intensity	% Positive Cancer Cells	Intensity
**OV11**	10	low	30–35	strong	<1	NA	40	int./strong	50–60	strong
**OV12**	50–55	low	<1	NA	<1	NA	35	strong	60	strong
**OV13**	<1	NA	5–10	strong	<1	NA	20	int.	40	strong
**OV14**	90	strong	5–10	int.	<1	NA	50–60	strong	70–80	strong
**OV15**	65	int.	<1	NA	<1	NA	40	strong	50	int.
**OV16**	60	low	<1	NA	50	low	30–40	int./strong	<1	NA
**OV17**	30–40	low	<1	NA	<1	NA	20	strong	40	int.
**OV18**	70–80	low	<1	NA	5	low	60	strong	70–80	strong
**OV19**	90	Int.	<1	NA	70–80	low	50–60	strong	70–80	strong
**OV20**	50	low	1–5	strong	<1	NA	30	int.	50	strong
**OV21**	10	low	1–5	strong	60	low	20	strong	60	int.

<1: negative; NA: not applicable; int.: intermediate

**Table 2:** Ovarian cancer Tn-glycoproteins carrying the MGL binding motif.

Function	Gene	Protein	Glycopeptide Sequences	Glycan Moieties	Glycosylated Sites *
**Mucins**	MUC1	Mucin-1	SGHASSTPGGEKETSATQR	4	S6; T7; T14; S15
			SSTPGGEKETSATQR	4	S2; T3; T10; S11
	MUC16	Mucin-16	FLHSEMTTLMSR	2	T7; T8
			IHPSSNTPVVNVGTVIYK	4	S4; S5; T7; T14
			ISTPDHDKSTVPP	4	S2; T3; S9; T10
	MUC24	Mucin-24	VTTPAPETCEGR	2	T2; T3
**Protein folding/cell metabolism**	ERP44	Endoplamic Reticulum Resident Protein 44	EFHHGPDPTDTAPGEQAQDVASSPPESSFQK	2	S22; S23
	LAMP1	Lysosome-associated membrane glycoprotein 1	CEQDRPSPTTAPPAPPSPSPSPVPK	5	T9; T10; S17; S19; S21
			RPSPTTAPPAPPSPSPSPVPK	5	T5; T6; S13; S15; S17
	LAMP2	Lysosome-associated membrane glycoprotein 2	TSTVAPTIHTTVPSPTTTPTPK	7	T3; T7; T10; T11; T16; T17; T18
	QSOX1	Sulphydryl oxidase 1	EAAQTTVAPTTANK	4	T5; T6; T10; T11
**Protein folding/cell metabolism**	SEL1L	Protein Sel-1 homolog 1	TTLTSDESVKDHTTAGR	2	T13; T14
	LRR8CD	Leucin-rich repeated-containing protein 8D	AHTPPGNAEVTTNIPK	3	T3; T11; T12
**Matrix/cell interaction**	AGRN	Agrin	ATTASRLPSSAVTPR	4	T2; T3; S10; T13
			APHPSHTSQPVAKTTAAPTTR	5	T7; S8; T14; T15; T19
	APP	Amyloid beta A4 protein	GLTTRPGSGLTNIK	3	T3; T4; T11
	DAG1	Dystroglycan	IRTTTSGVPR	4	T3; T4; T5; S6
	FN1	Fibronectin	HTSVQTTSSGSGPFDVR	2	T6;T7
	NID-2	Nidogen-2	SYPASGHTTPLSR	2	T8; T9
	PODXL	Podocalyxin	SSHSVTTDLTSTK	7	S2; S4; T6; T7; T10; S11; T12
	SDC3	Syndecan-3	LVSTATSRPR	3	S3; T4; S7
			TPTPETFLTTIR	3	T6; T9; T10
	VCAN	Versican core protein	HLVTTVPKDPEAAEAR	2	T4; T5
			YLSTTPFPSQHR	2	T4; T5
			QESSTTFVSDGSLEKHPEVPSAK	3	T5; T6; S21
			STILPTAEVEGTKAPVEKEEVK	4	S1; T2; T6; T12
			STILPTAEVEGTKAPVEK	4	S1; T2; T6; T12
			LHTTSAFKPSSAITK	4	T3; T4; S11; T14
			RKEEEGTTGTASTFEVYSSTQR	4	T7; S12; S18; S19
			TTDYSVLTTKK	4	T1; T2; T8; T9

Tn-glycopeptide sequences were found in both tumor and ovarian cell cancer cell lines. *: T and S: threonine and serine amino acid residues, respectively.

## Supplementary Materials

The following are available online at https://www.mdpi.com/2072-6694/12/10/2841/s1, Figure S1: Evaluation of MGL’s ability to bind STn glycopeptides. Figure S2: Characterization of COSMC deletion in SKOV-3 SC and rhMGL and VVA lectin binding in both parental and isogenic SKOV-3 and OVCAR-3 cell lines. Figure S3: The O-glycoproteome derived from tumor cells. Figure S4: Selected mass spectra showing enrichment of Tn-peptides. Figure S5: Mass spectra of (a) IGFBP7, (b) NUC1 and (c) ERp44 glycopeptides. Table S1: Peptide and glycopeptides used for MGL–LWAC set up. Table S2: Patients’ characteristics. Table S3: Lists of glycopeptides isolated by MGL- and VVA–LWAC from ovarian cancer SKOV-3 SC and OVCAR-3 SC (secretome or TCL). Table S4: Lists of glycopeptides isolated by MGL- and VVA–LWAC from tumor samples OV1,2 and OV3,4, respectively. Table S5: Accumulated data listing the glycoprotein identified in ovarian cancer OVCAR-3 SC and SKOV-3 SC cell lines and ovarian tumor samples
