# Supplementary material for: Investigating Patterns of Immune Interaction in Ovarian Cancer: Probing the O-glycoproteome by the Macrophage Galactose-Like C-Type Lectin (MGL)
Source: Cancers (Basel). 2020 Oct 1;12(10):2841. doi: 10.3390/cancers12102841 (PMC7600217; doi:10.3390/cancers12102841)
Supplement: Supplementary file 1 [file cancers-12-02841-s001.zip › cancers-908282-supplementary materials.pdf]

Supplementary Material: Investigating patterns of immune interaction in ovarian cancer: probing the O-glycoproteome by the Macrophage Galactose like C-type Lectin (MGL)

Chiara Napoletano, Catharina Steentoff, Federico Battisti, Zilu Ye, Hassan Rahimi, Ilaria Grazia Zizzari, Marco Dionisi, Bruna Cerbelli, Federica Tomao, Deborah French, Giulia d'Amati, Pierluigi Benedetti Panici, Sergey Vakhrushev, Henrik Clausen, Marianna Nuti and Aurelia Rughetti

Table S1. Peptides and Glycopeptides utilized in the study.

Peptide	Amino Acid Sequence	M.W.
IgA	VPSTPPTPSPSTPPTSPSK	1960.4
PDPN	VAMPGAEDDVVTPGTSEDRY	2109.9
1Tn- PDPN	VAMPGAEDDVVT*PGTSEDRY	2312.9
1T- PDPN	VAMPGAEDDVVT*PGTSEDRY	2474.9
5Tn-IgA-H	VPSTPPTPSPSTPPTSPSK§	2975.9
6Tn-IgA-H	VPSTPPTPSPSTPPTSPSK§	3201.8
7Tn-IgA-H	VPSTPPTPSPSTPPTSPSK§	3404.8

PDPN: Podoplanin; IgA-H: heavy chain IgA; * Glycosylated sites; §Ambiguous glycosylation sites.

Table S2. Patients' characteristics.

Patient	Age at Diagnosis	Histology	Stage
**OV 01**	60 years	HGSOC	IIIC
**OV 02**	40 years	HGSOC	IIIC
**OV 03**	68 years	HGSOC	IIIC
**OV 04**	75 years	HGSOC	IIIC
**OV 11**	53 years	HGSOC	IIIB
**OV 12**	63 years	HGSOC	IC
**OV 13**	65 years	HGSOC	IIIB
**OV 14**	81 years	HGSOC	IIIA
**OV 15**	59 years	HGSOC	IIIC
**OV 16**	48 years	HGSOC	IVB
**OV 17**	32 years	HGSOC	IVB
**OV 18**	82 years	HGSOC	IIIC
**OV 19**	80 years	HGSOC	IIIC
**OV 20**	67 years	HGSOC	IIIC
**OV 21**	61 years	HGSOC	IIIC

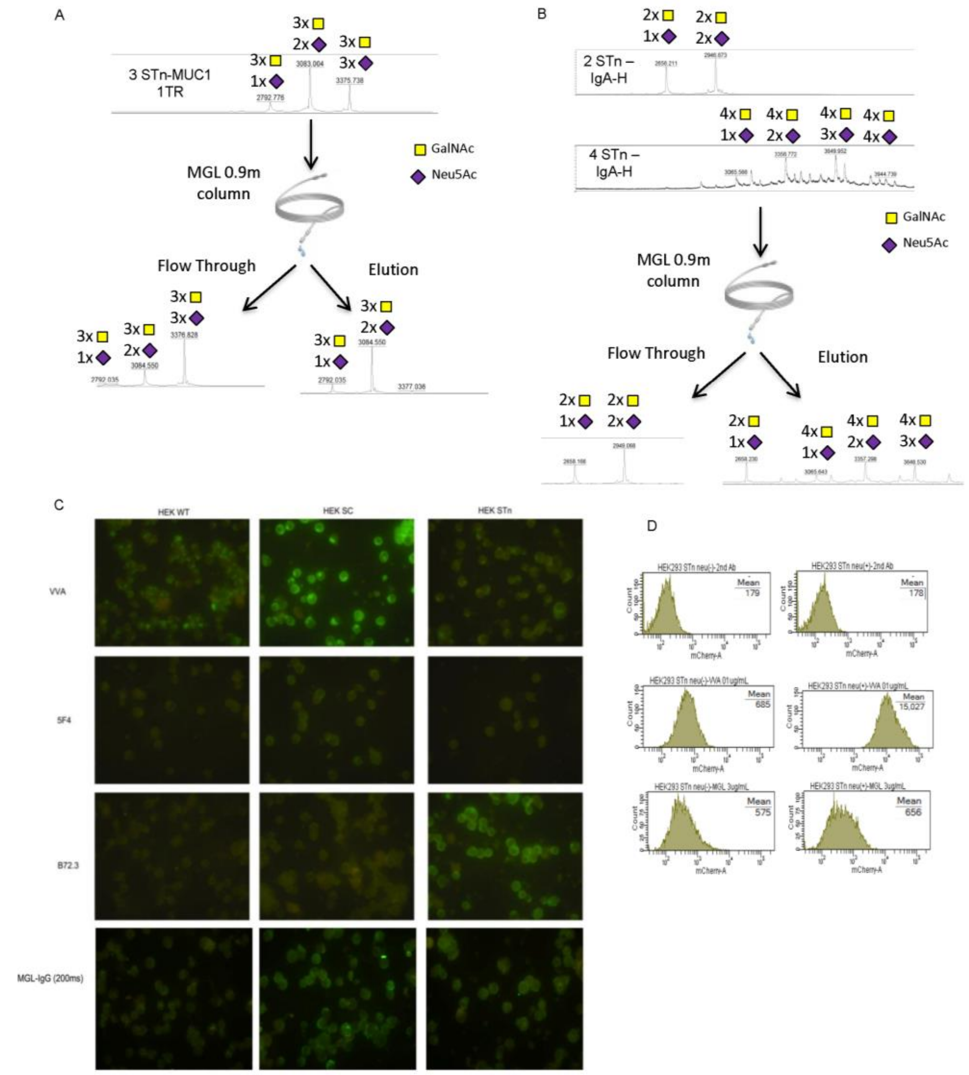

Figure S1. Evaluation of the ability of macrophage galactose like C-type lectin (MGL) to bind STn glycopeptides. (**a**) rhMGLLWAC carried out with a fully STn-glycosylated MUC1-1TR and with a mixture of TnandSTn-carrying MUC1-1TR glycopeptides. (**b**) rhMGL-based lectin weak affinity chromatography (LWAC) performed with a mixture of fully STn-glycosylated IgA-H peptide and with a mixture of Tn- and STn carrying IgA-H peptides. (**c**) Immunofluorescence microscopy of wild type HEK293(HEK293 WT) (first column), SimpleCell modified HEK293 (HEK293 SC) (second column) and STn-expressing HEK293 (HEK293 STn) (third column). Cells were stained with biotin *Vicia villosa* agglutinin (biotin-VVA) (first line), mAb 5F4 (second row, anti-Tn) and mAb B72.3 (third row, anti-STn) and rhMGL (fourth row). (**d**) Flow cytometry of HEK293 STn without neuraminidase treatment (first column) and with neuraminidase treatment (second column) stained with an Isotype Cy3 control (first row), biotin-VVA (second row) and rhMGL (third row).

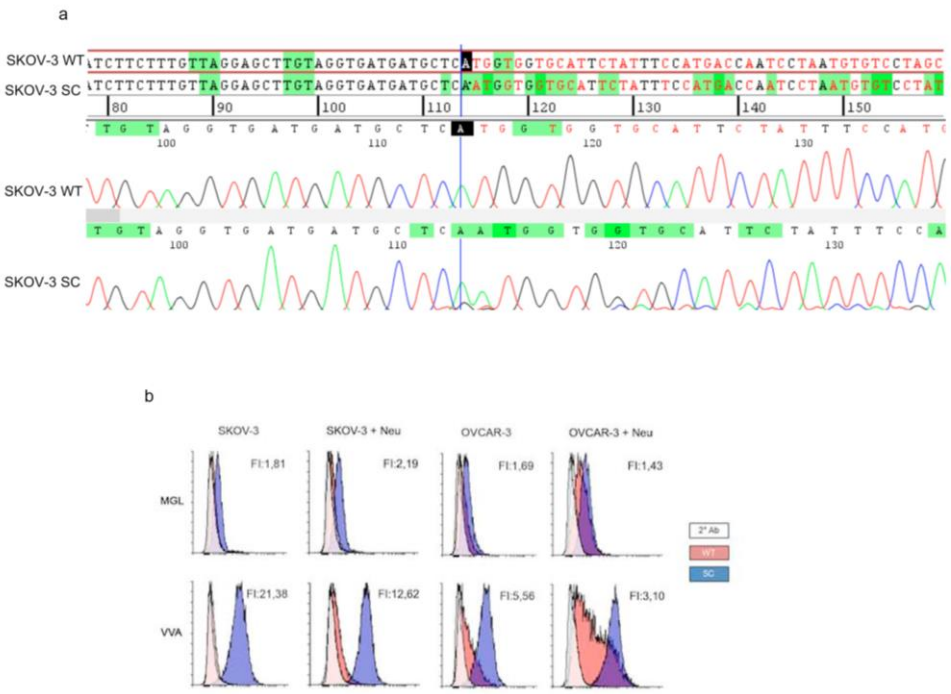

Figure S2. Characterization of COSMC deletion in SKOV-3 SC and recombinant human macrophage galactose like C-type lectin (rhMGL) and *Vicia villosa* agglutinin (VVA) lectin binding in both parental and isogenic SKOV-3 and OVCAR-3 cell lines. (**a**) Sequencing of PCR-amplified COSMC gene on SKOV-3 after CRIPR/Cas9 treatment revealed +1 and −2 biallelic mutation. (**b**) FACS analysis of wild type or glycoengineered SKOV-3 SC and OVCAR-3SC with and without neuraminidase treatment. FI: fold increase between the mean fluorescence intensity (MFI) of SimpleCell and MFI of the wild-type cells.

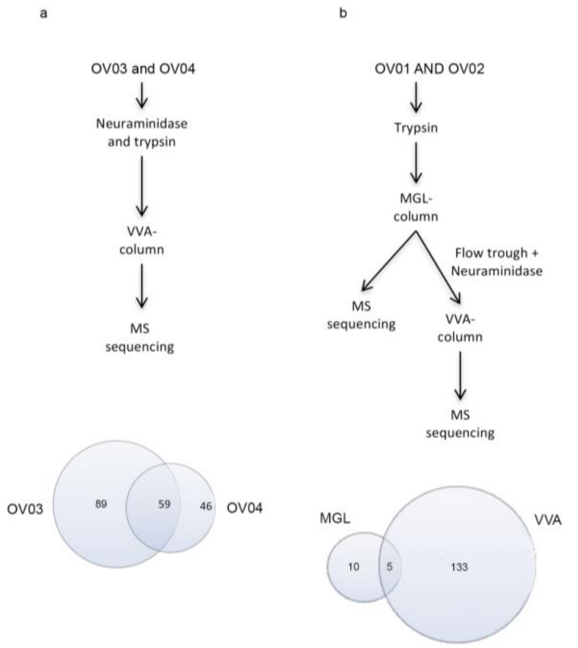

Figure S3. The *O*-glycoproteome derived from tumor cells. (**a**) Schematic depiction of the *Vicia villosa* agglutinin- lectin weak affinity chromatography (VVA-LWAC) strategy applied for the identification of the *O*glycoproteome of OV03 and OV04 together with the number of glycoproteins obtained in the two tumors. (**b**) Schematic representation of the macrophage galactose like C-type lectin (MGL) and

VVALWAC strategy used for the identification of the Tn-glycopeptides of OV01 and OV02. Diagrams depict the number of Tn-glycoproteins identified from OV01 and OV02 tumors.

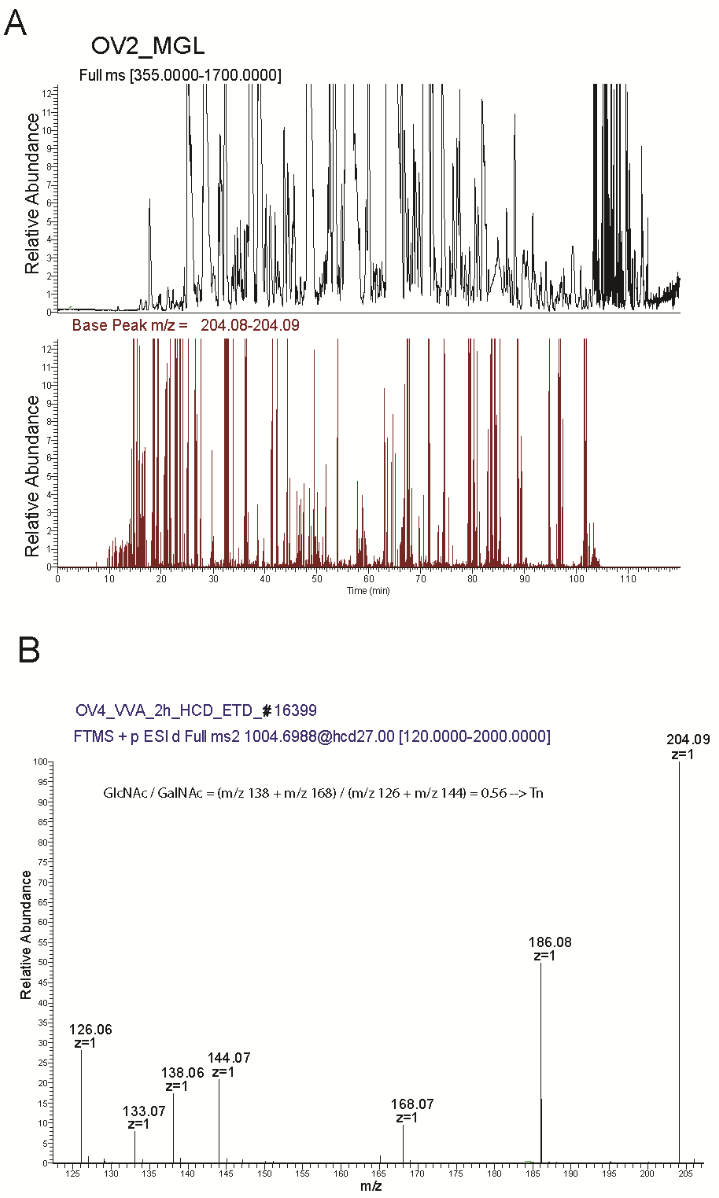

Figure S4. Selected mass spectra showing enrichment for Tn-peptides. (**A**) Full scan (upper panel) and extracted ion chromatogram (lower panel) for a macrophage galactose like C-type lectin (MGL) run (OV2_MGL).(**B**) Oxonium ions of a MS2 spectrum in a *Vicia villosa* agglutinin (VVA) run (OV4_VVA). The GlcNAc/GalNAc ratio indicates the identification of a Tn-peptide [35].

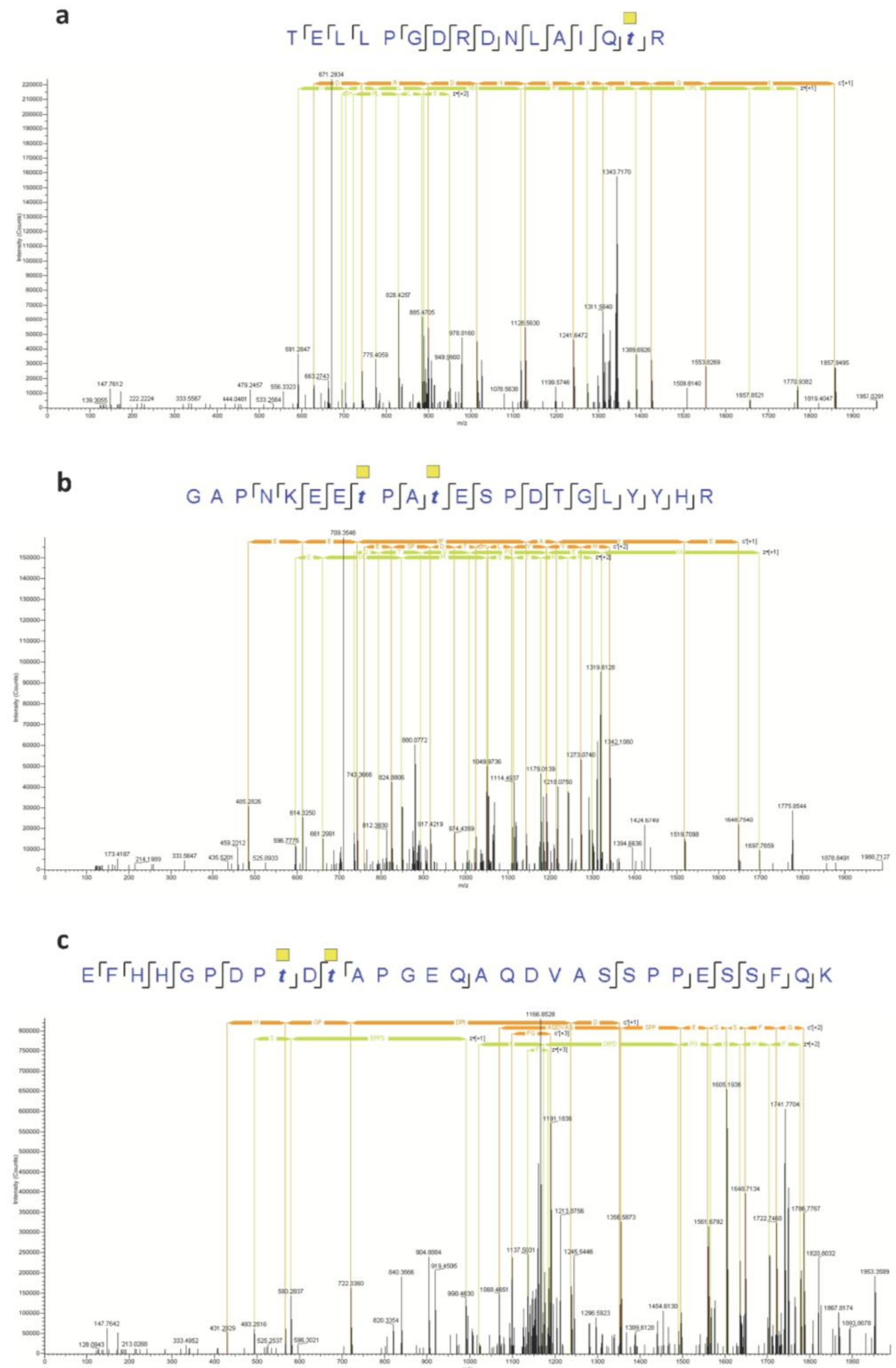

Figure S5. Mass spectra of (**a**) IGFBP7, (**b**) NUC1 and (**c**) Erp44 glycopeptides. This consensus view displays c and z fragment ion series in the spectrum and fragmentation ion coverage notation. The ┘ flag in the fragment ion coverage notation represents the c ions and the flag represents the z ions. Unmodified amino acids are displayed in capital letters and modified amino acids are displayed in lowercase letters.

© 2020 by the authors. Licensee MDPI, Basel, Switzerland. This article is an open access article distributed under the terms and conditions of the Creative Commons Attribution (CC BY) license (http://creativecommons.org/licenses/by/4.0/).
